# A review of bismuth‐based nanoparticles and their applications in radiosensitising and dose enhancement for cancer radiation therapy

**DOI:** 10.1049/nbt2.12134

**Published:** 2023-05-04

**Authors:** Daryoush Shahbazi‐Gahrouei, Yazdan Choghazardi, Arezoo Kazemzadeh, Paria Naseri, Saghar Shahbazi‐Gahrouei

**Affiliations:** ^1^ Department of Medical Physics School of Medicine Isfahan University of Medical Sciences Isfahan Iran; ^2^ School of Medicine Isfahan University of Medical Sciences Isfahan Iran

**Keywords:** nanomedicine, nanoparticles, radiation therapy

## Abstract

About 50% of cancer patients receive radiation therapy. Despite the therapeutic benefits of this method, the toxicity of radiation in the normal tissues is unavoidable To improve the quality of radiation therapy, in addition to other methods such as IMRT, IGRT, and high radiation dose, nanoparticles have shown excellent potential when ionising radiation is applied to the target volume. Recently, bismuth‐based nanoparticles (BiNPs) have become particularly popular in radiation therapy due to their high atomic numbers (Z), high X‐ray attenuation coefficient, low toxicity, and low cost. Moreover, it is easy to synthesise in a variety of sizes and shapes. This study aimed to review the effects of the bismuth‐based NP and its combination with other compounds, and their potential synergies in radiotherapy, discussed based on their physical, chemical, and biological interactions. Targeted and non‐targeted bismuth‐based NPs used in radiotherapy as radiosensitizers and dose enhancement effects are described. The results reported in the literature were categorised into various groups. Also, this review has highlighted the importance of bismuth‐based NPs in different forms of cancer treatment to find the highest efficiency for applying them as a suitable candidate for various cancer therapy and future clinical applications.

## Graphical abstract

This review has highlighted the importance of bismuth‐based nanoparticles in different forms for cancer treatment to find the highest efficiency for applying them as a suitable candidate for various commercial and future clinical applications due to their dose enhancement properties, radiosensitisation, and superior biocompatibility.

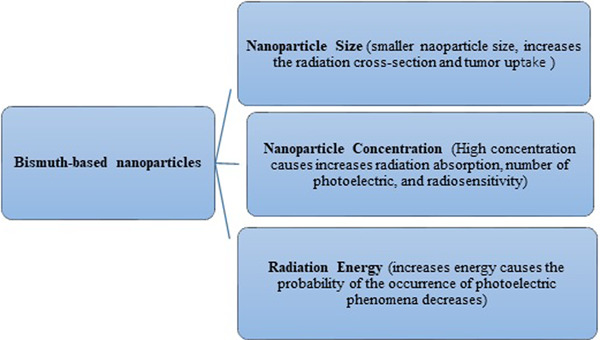

## 1 INTRODUCTION

Cancer is one of the important leading causes of death worldwide. By 2030, it is estimated that there will be approximately 22 million new cases of cancer worldwide and 13 million deaths from cancer [1, 2]. Clinically, the most common cancer treatments include surgery, chemotherapy, and radiation therapy, which are the main non‐invasive treatments for many of these types [3]. For this purpose, radiotherapy uses ionising radiation, but unfortunately, due to hypoxia, tumour cells are more resistant to radiation than normal cells. This can lead to treatment failure and can also cause side effects in normal tissues [4]. Hence, the use of radiosensitizers may be beneficial to enhance the delivery of radiation doses to tumour cells [5].

Recently, as a new radiosensitising technique in medicine, nanoparticles (NPs) may have improved the treatment of various types of tumours [6]. Therefore, many studies are limited to in vitro and in vivo conditions of nanoparticle application in radiotherapy [7, 8]. However, the selection of NPs as radiosensitizers is difficult. Bismuth nanoparticles (BiNPs) with specific properties such as high atomic numbers (Z), high X‐ray attenuation coefficient, low toxicity, and low cost can be used as radiosensitizers to increase uptake by many cancer cells and increase absorption of radiation in synergistic cancer therapy [9]. It is also important to note that bismuth oxide and other bismuth‐based compounds as nanoparticles are highly biocompatible and have low toxicity. Moreover, it is inexpensive compared to other high‐Z materials such as gold and platinum [10]. Although bismuth is one of the heavy metals on the periodic table, bismuth can be easily synthesised in a variety of sizes and shapes and can be coated on surfaces with appropriate biological functions [11]. Due to advances in medicine, today's efforts aim to use targeted compounds to treat cancer [12].

Therefore, investigation the use of bismuth‐based NPs (targeted or non‐targeted) in radiotherapy is a promising approach to improve the efficiency of cancer treatment. The methodology used in this literature review were focussed on the articles in databases including Google Scholar, PubMed, Scopus, ProQuest, and Web of Knowledge from December 22, 2019 until March 2023.

The review article aimed to describe the strategies for applying bismuth‐based NPs and its chemical drug‐loaded for enhancing cancer cells' sensitivity to radiation and radiation dose enhancement. Then, it is attempted to present the recent studies on targeted and non‐targeted bismuth‐based NPs as new radiosensitiser and dose deposit nanoprobes for better treatment in various platform of cancer therapy.

### 1.1 Non‐targeted nanoparticles

Nowadays, the utility of bismuth‐based NPs as a powerful theranostics agent to enhance the efficacy of radiation therapy for cancer treatment and to improve the resolution of different imaging modalities for cancer diagnosis is considered by many researchers [7]. Several studies have shown that Bi NPs can sensitise tumour cells by enhancing reactive oxygen species (ROS) and mitigating hypoxia. The study admitted by Stewart et al., is the first evidence of the use of bismuth oxide nanoparticles as effective radiosensitizers for highly radioresistant cancer cells. They concluded that in the presence of 125 kV and 10 MV beams, there was a good dose‐enhancing effect, with sensitisation enhancement ratio (SER) of 1.48 and 1.25 respectively [13].

In a study, Sisin et al. [14] also investigated the radiosensitivity effects of BiO (bismuth oxide) NPs using electron and photon beams on MCF‐7, MDA‐MB‐231, and NIH/3T3 cell lines. Using a concentration of 0.5 μM as the highest concentration did not cause high oxidative stress in the cell and a concentration of 0.5 μM and a photon energy of 10 MV resulted in a SER of 2.29.

In another work, Rashid et al. [15] demonstrated the radiobiological effects of bismuth oxide nanorods (BiONR), platinum nanodendrites (PtND), superparamagnetic iron oxide nanoparticles (SPIONs), and gold nanoparticles (AuNPs) on the HCT116 cell, which irradiated with a 150 MeV proton beam. The highest value of SER was associated with bismuth oxide nanorods, then platinum nanodendrites, followed by gold, and finally superparamagnetic iron oxide nanoparticles. In their studies the SER levels found to be 4.93, 3.08, 2.64, and 1.95 respectively.

In this line, Liu et al. [16] demonstrated that a nanoradiosensitiser called bismuth oxide (BiO_2_‐x) nanosheet could improve radiation efficiency by generating ROS to kill cancer cells and alleviate tumour hypoxia. Their results showed that increasing the concentration of BiO_2_‐x Ns increased the O_2_ content.

In a study by Ma et al. [3] 190 nm diameter bismuth nitrate and cisplatin were investigated for radiosensitivity and DNA damaging activity as suitable radiosensitizers. They found that when nanoparticles internalised by tumour cells, bismuth in NP@PVP (Polyvinylpyrrolidone) stimulated radiotherapy (RT) by increasing the amount of ROS and enhancing DNA damage after X‐ray irradiation. Cisplatin, on the other hand, inhibits DNA damage repair. Their findings demonstrated a superior anti‐cancer potential in synergistic chemo‐radiotherapy using NP@PVP nanoparticles. Cisplatin, on the other hand, inhibits DNA damage repair. Their findings demonstrated a superior anti‐cancer potential in synergistic chem‐oradiotherapy using NP@PVP NPs.

Furthermore, Sisin et al. [17], reported that bismuth oxide nanoparticles (BumPs), cisplatin (Cis), and baicalein‐rich fraction (BRF) have the synergistic effect. They also illustrated the effects of natural‐based agent on ROS under irradiation by photon, electron and high dose rate (HDR) brachytherapy of clinical radiotherapy beams on breast cancer and normal fibroblast cells. Their reported that ROS generated in the presence of Cis stimulated the most significant amount of ROS compared to BiONPs and BRF. However, the combination of the components resulted in higher ROS levels for photon beam compared to electron beam and brachytherapy. But, the radiosensitising effect of brachytherapy was more pronounced compared to photon beams and electron beams.

Similarly, in 2020 Gao et al. [18] investigated the radiosensitisation effect of Bi_2_S_3_‐MoS_2_ nanoparticles (BM NPs). Their results demonstrated that these nanoparticles can act as efficient nanoradiosensitizers through dual roles. Sufficient heating increases the X‐ray attenuation coefficient. These results may be attributed to enhanced tumour oxygenation and ROS effects and the self‐repair capacity of cancer cell DNA.

Farahani et al. [19] have vinvestigated the quantification of dose enhancement factors using Bi NPs compared with Au‐based nanoparticles (AuNPs) at different clinical energies. In their wok, the samples exposed to iridium‐192 and cobalt‐60 and they have shown that BiNPs combined with brachytherapy sources has the ability to improve the effectiveness of radiation therapy for cancer treatment.

A nano radiosensitiser BiO_2_‐x nanosheets study was performed by Liu et al. [16] in which they demonstrated it could improve radiation efficiency by inducing ROS to kill cancer cells and at the same time reduce tumour hypoxia. Indeed, their results showed that the O_2_ content increased with increasing concentration of BiO_2_−x NSs.

In the study of Qin et al. [20], the ability of Bi‐based mesoporous upconversion nanophosphor loaded with doxorubicin (UCNP‐DOX) on the specific anti‐tumour immune effects of X‐ray radiotherapy has been evaluated both in vitro and in vivo. They found that tumour‐associated macrophages (TAMs) are highly radioresistant to ionising radiation. In their work, the TAMs repolarisation and tumour growth inhibition reported for the first time. In addition, they claimed that UCNP‐DOX had lower side effects than the DOX group alone because it prevented the fur loss of mice, while the second group showed severe hair loss in the mice.

Abhari et al. [21] successfully investigated the tumour radiosensitising ability of Bi_2_S_3_‐Au‐BSA‐FA hybrids in vitro and in vivo. They concluded that mice survival were improved. Indeed, the secondary electron cascade and Schottky barrier production can enhance the treatment efficiency of radiotherapy.

However, in another paper published in 2020, Zhang et al. [22] studied the effect of novel BiPt‐folic acid‐modified amphiphilic polyethylene glycol (PFA) composite nanosystems as a radiosensitizers to enhance the absorption of X‐rays at the tumour sites. BiPt‐PFA can reduce hypoxia in tumours. As the concentration of BiPt‐PFA increased, the effects of hypoxia decreased. In addition, the application of an 808 nm laser had an effect on cell proliferation because X‐ray coherence in cells incubated with BiPt‐PFA reduced the survival rate to 11%. More studies and their details in this line are listed in Table 1.

**TABLE 1:** Bismuth‐based NPs and their characteristics in radiation therapy.

Reference	Nanoparticle	Concentration	Size (nm)	Energy	SER/DEF/effect
[13]	Bi_2_O_3_	50 (μg/ml)	50–70	125 kV 10 MV	1.48,1.25
[23]	BFO	(50, 100) μg/ml	5.5 ± 28	160 kVp	1.35,1.76
[14]	BiO	0.5 μM	60	10 MV	2.29
Jiao [24]	Bi	2 mg/mL	2–10		Tumour growth restriction
[25]	Bi_2_S_3_	(0.5,1) mM	3–5	80 kVp	DEF = 1.6,3.07
[26]	Bi_2_O_3_	0.05 µM	60	6 MV	3.45
[15]	BiNRs	1 mM	70	150 MV	4.8>
[9]	pBi coated with pvp	200 (μg/ml)	60		Significant increase in tumour cell death
[27]	Bi_2_O_3_‐PEG	0.5 µM	60	6, 10 e: 6,12 MeV	1.12, 1.15 1.13, 1.06
[28]	Bi_2_S_3_	100 μg/mL	10–70	‐	1.22
[29]	PVP‐Bi_2_Se_3_@Sec	50 ppm	29.2	160kV	1.5
[30]	BiO	0.5 mM	60	e: 0.38 MeV	1.70, 1.22, 0.66
[31]	BP/Bi_2_O_3_	1.2 μg/mL	300 ± 80	‐	Cell viability = %53.1
[32]	MnSe@Bi_2_ Se_3_ ‐PEG	40 μg/mL	132 ± 21 width 111 ± 21 nm	‐	1.6
[33]	BiOI QDs	50 (μg/ml), 2 mg/mL	5.51	‐	1.4 Significant tumour control
[34]	PVP‐Bi_2_WO_6_	100 μg/mL, 2 mg/mL	240	50 kV	Significant tumour growth inhibition
[35]	Bi_2_S_3_–PLGA	0–200 μg/mL	500–1000	‐	Obvious cell toxicity in cancer cells
[36]	PVP‐Cu_3_BiSe_3_	100 μg/mL, 4 mg/mL	13 ± 3	160 kV	1.51 Significant tumour growth inhibition
[37]	PVP‐Bi_2_Se_3_	200 μg/mL, 20 mg/kg	59.8	137Cs	Obvious enhancement in DNA damage
[38]	PEGylated Fe@Bi_2_S_3_	(0, 10, 50, 100) ppm	15	‐	Significant increase in apoptosis
[39]	BiGdO_3_	(50,100) μg/ml 6 mg/mL	11.3 ± 1.6	160 kV	Reduction in tumour volume
[40]	Bi_2_O_3_‐NR	0.5 μMol	70,80,90	6 MV	DEF = 1.38 2.20 2.83
[41]	BiONPs, Cis	0.5 mM	60	150 MeV	Proton radiosensitizers, ROS production
[16]	BiO_2−x_ nanosheets	0, 50, 100, and 200 μg/mL	150	160 kV	Generate adequate ROS
[20]	Bi‐based mesoporous loaded with doxorubicin	0–100 μg/mL	85	‐	Significant increase necrosis in tumour
[3]	NP@PVP + bismuth nitral cisplatin	2 mg kg−1	190	‐	Increasing radiosensitivity
[17]	BiO NPs‐Cis	0.5 mM	60	6 MeV 0.38 MeV iridium‐192	Increasing radiosensitivity
[42]	Bi_2_O_3_ NPs	46.596 μg/mL	60, 90	50 kV	Increasing radiosensitivity
[18]	Bi_2_S_3_‐MoS_2_	0, 25, 50, 100, 150, and 250 μg ml^−1^	50	‐	Increasing radiosensitivity
[19]	BiNPs	0.04 mg/mL	50	380 keV 1.25 MeV	Increasing radiosensitivity

### 1.2 Targeted nanoparticles

All of the following studies used bismuth as target nanoparticles in combination with radiation, and peptides and folic acid as target cellular receptors. For instance, Jie Liu et al. [43] have investigated Bi‐based nanospheres as radiosensitizers that investigated how to simultaneously achieve multimodal therapy and tumour microenvironment (TME). For this purpose, they used Bi@Mn with H_2_O_2_ responsive prodrug. It can be effectively loaded with docetaxel (DTX) and Bi@Mn‐DTX and further improved from folic acid modification and made Bi@Mn‐DTX‐PFA. The results of this study showed that Bi@Mn‐DTX‐PFA nanoparticles achieved multimodal therapy (radiotherapy, chemotherapy, and haemodynamic therapy) in hypoxic tumours.

In a study, Yu et al. [44] investigated the diagnostic and therapeutic functions of a peptide conjugate (LyP‐1) that can target cell surface P32 receptors. In this study, Bi‐LyP‐1 (targeted) and PEGylated Bi (non‐targeted) were used to investigate radiosensitisation in radiotherapy using various doses from 0 to 8 g in 4T1 cells. The amount of targeted NPs uptake by 4T1 cells was much higher than that of the non‐targeted nanoparticles. Also, no specific cytotoxicity was observed in 4T1 (cellular carcinoma) and L02 (normal) cells after 24 h incubation with nano‐complexes at concentrations ranging from 0 to 400 ppm. 4T1 cells were cultured for 24 h in the presence or absence of BiLyP‐1 NPs (10‐ppm bismuth concentration) for 24 h prior to irradiation and then irradiated with 0, 2, 4, 6, and 8 Gy of X‐rays. Then, the colony assay was performed SERD_0_ was obtained equal to 1.248.

In another study by Nosrati et al [45], they investigated the sensitising effect of bismuth sulfide nanoparticles using albumin‐coated alumina as a targeting molecule for direct binding to the tumour sites. In this study, they used nanoparticles with a size of 78.9 nm and concentrations of 25, 50 and100 μg/ml. Cellular uptake of nanoparticles induced by Bi_2_S_3_@BSA‐FA NPs was higher than Bi_2_S_3_@BSA NPs. Their results showed the cell viability of approximately 60% when the cells exposed to both targeted and untargeted NPs at a concentration of 100 μg/mL and a dose of 6 Gy. Further studies on the use of targeted nanoparticles in radiotherapy are presented in Table 2.

**TABLE 2:** Targeted bismuth‐based NPs in radiotherapy using x‐ray beam.

Reference	Nanoparticle	Surface/labelled	Concentration (μg/ml)	Size (nm)	Energy	SER\DEF\effect
[44]	Bi‐LyP‐1	LyP‐1	10 ppm	12	‐	SERD0 = 1.218
[46]	Bi_2_Se_3_ ‐CS‐RGD	RGD	(o to 160)	30	6 MV	Survival fractions decreases with dose and NP concentration
[47]	Bi	Folate‐RBC	(0–100)	46–56	kV	Survival fractions decreases with dose and NP concentration
[48]	Bi_2_S_3_	BSA‐FA	(25,50,100)	78.9	‐	Cell viability decreases
[22]	BiPt‐PFA	Folic acid‐modified amphiphilic polyethylene glycol	0–80	170	‐	Survival fraction was decreased
[21]	Bi_2_S_3_‐Au‐BSA‐FA hybrids	BSA‐FA	7.5, 75, 150	182.7	6 MV	Increasing radiosensitising
[49]	BSNPs	Lauric acid	20–60	25	‐	Increasing radiosensitivity

## 2 PROPERTIES OF BISMUTH‐BASED NANOPARTICLES

### 2.1 Nanoparticle size

One of the key factors affecting the sensitivity of nanoparticles is their size. This depends on how nanoparticles interact with radiation and biological systems. The nanoparticles should provide appropriate time for radiotherapy in the present of nanoparticles, while eliminate from the body after a certain time and accumulate in the organs like liver and heart, which renal clearance is the best achievable.

Typically, renal clearance of metal nanoparticles with hydrodynamic diameters less than 6 nm is independent of charge. This occurs for nanoparticles between 6 and 10 nm depending on charge, but occurs faster for positively charged nanoparticles than for negatively charged nanoparticles.

Also, large nanoparticles with a size of 10 nm diameter likely be captured by the liver. Smaller nanoparticles enriched in tumour tissue by diffusing from the bloodstream and thus distributed uniformly. Today's some studies noted to happen the most cellular uptake by large sizes probably 30 nm or more of NPs [50, 51].

Regarding the radiosensitivity of bismuth‐based NPs, some studies have concluded that the increase in SER and DEF (dose enhancement factor) is minimal for BiNPs less than 30 nm in diameter, although these the particles contribute to tumour susceptibility and cause an increase in tumour temperature. In addition, medium‐sized nanoparticles (30–70 nm) not only sensitise tumours, but also lead to enhanced ROS effects and duration of treatment, increasing free radicals in tumour cells and ultimately reducing survival ratio. It should be noted that SER and DEF are maximal in the large size nanoparticles (>70 nm) approximately two to four times more than that of small size. Large nanoparticles lead to inhibition of tumour cell proliferation and eventual death due to apoptosis, severe ROS, decreased colonisation, and increased free radicals in tumour tissue.

A study showed [27] that larger‐sized nanoparticles may be more effective in improving tumour susceptibility, but the amount deposited in body tissues due to their size may alter toxic effects. Although this is the minimum value for small nanoparticles and the maximum value for large nanoparticles, morphological consequences also affect toxicity, so it is difficult to define the toxicity mechanism according to the size of BiNPs needs further research. The size of the nanoparticles is also important when considering the interaction of nanoparticles with radiation. Therefore, the interaction of secondary electrons and radiation in the bulk of nanoparticles causes more ionisation events in large nanoparticles. Secondary electrons generated in larger nanoparticles were then more likely to release energy inside the nanoparticles before reaching the surface, thus reducing the radiation dose [51]. Therefore, the effects of generated free radicals are limited to the tumour volume. This may be another reason why larger nanoparticles are more effective at sensitising tumour cells.

According to the study of Abidina et al. [27], they showed that the size and shape of nanoparticles affected on its radiosensitisation. Except higher Z of bismuth, Bi_2_O_3_‐NP with high surface area has more potential to applied as radiosensitiser in barest cancer cells. Also, Alyani Nezhad et al. [42] investigated the size and shape of nanoparticles, and concluded that the nanoparticles with the largest size has the effective tumour radiosensitivity because the secondary electron's reabsorption chance is increased with nanoparticle size in the emitting Bi‐based NPs.

Hossain et al. [52] investigated the size of different nanoparticles. BiNPs with sizes from 2 to 400 nm were then shown to increase the Auger electrons absorption of the nanoparticles and reduce the dose of cells surrounding the nanoparticles NPs due to low‐energy and short‐range Auger electrons. On the other hand, the energy content of photoelectrons and characteristic X‐rays remains in the largest nanoparticles. As a result, the dose increase remains almost constant. The results of BiNPs are superior to other nanoparticles such as gold particles.

Moreover, Bulmahn et al. [10] showed that distribution of (BiO)_2_CO_3_ and (BiO)_4_CO_3_(OH)_2_ NPs in tumour cells for smaller NPs is greater than of largest of them. While, Khodadadi et al. [53] concluded there is a great difference between predicted nanoparticle size effect on dose enhancement and predicted it. They also showed that while increases uptake to tumour region for the larger size of nanoparticles, hence, it needed to more work to find the right balance but it was more useful in the depth of the tumour loaded.

Consequently, Zulkifli et al. [26] compared BiNPs in tow size 60 and 90 nm. They showed that Bi_2_O_3_ 60 nm is less toxic than 90 nm Bi_2_O_3_NPs to the MCF‐7 cells because the size of nanoparticles has critical roles in both the extent and rate of cellular uptake. They also showed that larger NPs have higher cellular uptake.

### 2.2 Nanoparticles concentration

One of the influencing factors to consider when choosing Bi‐based NPs is their concentration. Nanoparticle concentration related to the toxicity and intracellular uptake. Also, it was demonstrated that nanoparticle concentration could affect the formation of NPs with different sizes [27]. A study showed that increasing the concentration of nanoparticle resulted in an increase in DEF [25].

Moreover, recent studies have found that even concentrations as low as 0.1 mM of Bi_2_O_3_NPs during IORT (intraoperative radiotherapy) irradiation effectively increase the dose [42]. In another study it was established that a high concentration of NPs will be more radiosensitisation and also better radiotherapeutic effect on MCF‐7 and 4T1 cells [54].

Abhari et al. [21] concluded that the uptake rates of Bi_2_S_3_‐Au‐BSA and Bi_2_S_3_‐Au‐BSAFA hybrids increased with increasing their concentrations in 4T1 cells. Indeed, cell growth inhibition was greater with increasing concentrations of Bi_2_S_3_‐BSA.

Various studies confirmed the low cytotoxicity of bismuth NPs both in vitro and in vivo [18, 45, 55]. For instance, it was shown that Bi_2_S_3_ nanorods and BMNPs have no noteworthy cytotoxicity up to a concentration of 200 and 250 μg mL^−1^, respectively, indicating that bismuth nanomaterials could be safely applied in biological applications.

Sisin et al. [56] reported that a concentration of 0.5 mM BiONPs observed as a suitable concentration to use for radiosensitisation of various cell lines without toxic effects. However, in vitro studies demonstrated that the concentration of BiNPs in the presence of X‐rays could affect cytotoxicity [24].

Recently, cell viability assessed under normoxic and hypoxic conditions using media containing BiPt‐PFA. The results  showed that the effect of hypoxia decreased as the concentration of BiPt‐PFA increased [22]. Recent studies have shown that increasing concentrations of T‐BiO_2_‐x NSs and Bi@Mn‐PFA lead to increased O_2_ and ROS production and a gradual decrease in cell viability [16, 43].

### 2.3 Radiation energy

Radiotherapy is a vital modality in cancer treatment that utilises different kind of radiation such as photon and electron beams to kill the cancer cells in various depth. Although the advantages of the radiosensitising effect when combining Bi_2_O_3_‐PEG NPs with photon and electron beams have been demonstrated, they have different radiation interaction mechanisms [27].

Advanced radiotherapy techniques such as intraoperative radiotherapy (IORT) have achieved DEFs of approximately 3.28 and 2.50 for planar and spherical Bi_2_O_3_ NPs respectively. This is the result of amplifying photoelectric effect by declining energy up to 50 kV range in the IORT as the photoelectric effect is the principal procedure for photons with energy from 10 to 500 keV [42].

Farahani et al. [19] reported that the dose enhancement about 16.35% and less than 4% under exposure of the cobalt‐60 source and Iridium‐192, respectively for BiNPs using the MRI polymer gel dosimetry technique which indicates the relation between energy and dose enhancement.

In a study by Rashid et al. [15] the radiosensitisation effects of different nanoparticles irradiated with 150 MeV proton beams was assessed. They observed that BiNRs had the highest ROS generation and sensitisation enhancement ratio (SER) equal to 4.93 among other materials.

Another study [56] evaluated the relationship between photon beam energy and DEF in the presence of BiONPs in the range of 0.001–20 MeV. The results show that the highest DEF for BiONPs is 4.51 at 0.04 MeV. In fact, DEF is highly dependent on beam energy.

### 2.4 Physico‐chemical properties and their figure in radiosensitisation coated

Coating of nanoparticles is a key factor in nanoparticle applications, as the coating of nanoparticles can control the interactions between nanoparticles and proteins in the bloodstream [57, 58]. New BiNPs have developed as excellent nanoplatforms for drug delivery, CT imaging, radiotherapy, and chemo‐photothermal therapy. For this reason, the biocompatibility of nanoparticles is very important. In this study, by reviewing the studies, it found that they use various coatings for the surface of nanoparticles. Ma et al. obtained pBi nanospheres have good biocompatibility. And they mentioned the reason is due to PVP coating the nanoparticles NPs surface [9]. Jiangfeng et al. used Bi_2_Se_3_NPs in radiotherapy and reported that the use of PVP coating can increase the biocompatibility of nanoparticles [29].

Also, in a study Jiangfeng et al. have shown that in the use of Cu_3_BiSe_3_ NPs, coating them with PVP could significantly improve the stability of Cu_3_BiSe_3_ NPs in the physiological solution and their biocompatibility [36].

In another study, Abidin et al. use PEG (polyethylene glycol) coated bismuth oxide nanorods for inducing radiosensitisation on MCF‐7 breast cancer cells under irradiation of megavoltage radiotherapy beams. The described surface coating can be applied to reduce nanoparticle toxicity and generate biocompatible nanoparticles [27]. In both in vitro and in vivo experiments, the surfaces of nanoparticles are mostly modified with polymers such as PEG, polyvinyl alcohol PVA, and poly lactide‐co‐glycolic acid (PLGA) [59, 60]. Neutral polymers are excellent biomedical materials used to reduce the systemic toxicity of nanoparticles, and these polymers increase circulation time and stability. In a study by Rajae et al. the surface of the nanoparticles was modified with PEG to achieve good biocompatibility [54].

Additionally, nanoparticles coatings are used to specifically target tumour cells in the body (active targeting). Targeting strategy employed to ensure adequate concentration of nanoparticles in tumour cells [61]. In contrast, targeted drug strategies limit the distribution of drugs to specific organs, thus offering the opportunity to reduce side effects and improve efficacy. In active targeting, nanoparticles are functionalised with special molecules that interact with receptors known to be selectively agreeable to tumour cells [62]. The major stimulant is to relying on passive uptake through the enhanced permeability and retention (EPR) effect [63]. This has been attained, for example, with antibodies [64], peptides [65], folates [66], and aptamers [67].

## 3 DISCUSSION

This study investigated Bi‐based NPs literature and only a few of them investigated the effects of BiNPs in combination with other sources except photon beams with various energy like proton and electron [15, 17, 19, 27, 41].

The results show that non‐coated nanoparticles compared to nanoparticles with surface coating in the same physical properties and concentrations have different dose effects in the same cells. It also showed that the nanoparticles with a coating (like PEG) created less cellular toxicity. This indicates the toxicity of BiNPs can reduced by coating their surface. Therefore, it will enable to the use of nanoparticles at higher concentrations and achieve higher SER.

From point of NPs size, researchers concluded that smaller NPs have been shown to be more likely to penetrate tumours, although this ability varies by type of NPs [68]. To accumulate in the tumour, the smaller size nanoparticles are used because they are appropriate for blood circulation [69]. Moreover, the larger surface‐to‐volume ratio of smaller NPs increases the radiation cross‐section and also increases tumour uptake [70]. Also, the self‐absorption of the secondary electron in larger NPs is more prominent compared to small sizes [42].

One of the factor that affects SER is the energy of the radiation used in radiotherapy. The results showed that a higher SER value was obtained under the same conditions (same cell line and the same nanoparticles) when using less radiant energy beams compared with high energy beams at lower concentrations of nanoparticles. This is probably because, with increasing energy, the probability of the occurrence of photoelectric phenomena decreases (it is related to energy^−3^), and as a result, the rate of absorption of the incoming beam decreases. The highest SER obtained when high‐energy proton beams used. In this case, the amount of energy transferred is very high due to the very high LET of the protons, and this is an important factor in radiosensitising to kill the tumour cells. Other types of radiation, such as photons, electrons, and gamma rays, are also used to radiosensitise in the presence of nanoparticles, but the use of X‐rays is more common and in the same condition due to the type of interaction with NPs.

Another important factor to consider is concentration. After observing the above studies, it appears that with increasing the concentration of nanoparticles, the amount of radiation absorption increases and as a result, the number of photoelectrons and Auger electrons will be increased. Therefore, the degree of radiosensitivity by the use of nanoparticles increases. However, it should be noted that the concentration can not be increased freely because it causes toxicity. The concentration at which cytotoxicity occurs depends on several factors such as nanoparticle type, nanoparticle size, and nanoparticle coating.

Among BiNPs, the use of bismuth oxide NPs is very common. Hence, the most studies have evaluated the radiation sensitivity of this nanoparticle, which is relatively more effective because bismuth has a very high atomic number (*Z* = 83) and is the heaviest metal element of the periodic table. That is reason why it can increases the probability of photoelectric effect compared to other metal elements. When using nanoparticles based on bismuth by combining elements such as S, Se, and Gd, due to their lower atomic number when exposed to the ionising radiation, they interact with the radiation resulting in a decrease in the production of photoelectrons and Auger electrons. For this reason, they leads to a decrease in the radiosensitivity generated compared to using of bismuth nanoparticles. Therefore, using of BiNPs, they can provide a good radiosensitivity for destroying the tumour cells and less toxicity.

An important and effective factor in using nanoparticles for specific radiosensitisation at the tumour site is the use of these targeted NPs in radiation therapy. For instance, in a study by Deng et al. [47] they used targeted and non‐targeted BiNPs and the survival fraction was almost as low as half the survival fraction when using bismuth targeted with folate NPs versus using non‐targeted BiNPs. This is because there are many folate receptors on the surface of cancer cells and it is a good feature due to increase in the number of cancer cells uptake from folate‐targeted bismuth through folate receptors. By increasing the cancer cells' that uptake a nanoparticle, absorption of radiation increases and leads to the production of more reactive oxygen species (ROS), photoelectrons, and Auger electrons, resulting in more single‐stranded and double‐stranded DNA damage. All of these increases the radiosensitivity and ultimately leads to more cell death. Several studies have focussed on the mechanism of Bi‐based NP‐induced radiosensitivity enhancement [13, 14, 15, 71].

For instance, Stewart et al. [13] highlighted that BiONPS could be tailored with various oxygen contents, inducing cell proliferation or toxicity. As stated in the literature, the presence of oxidative stress produced by the NPs in the cell would produce free radicals that will possibly lead to damage to the DNA in the cells [72]. Also, Sisin et al. [71] work results showed the intercellular generation of ROS due to the presence of BiONPs. After treatment with different sizes and different concentrations of BiONPs on the NIH/3T3 cells, they reported that the formation of ROS is not affects by the size and concentration of NPs. They indicated that ionising radiations might disrupt the cancer cells' progression either by direct radiation energy or by the mechanism of ROS indirect and incremented cell killing. In another study, Abdul Rashid et al. [15] indicated that ROS generation is normally correlate with the level of radiosensitisation and plays a fundamental role in the radiosensitisation mechanism. The ROS results demonstrated that BiNRs produced the highest ROS compare to PtNDs, AuNPs, and SPIONs. The decrease in cell survival dependent to the high level of ROS produced by respective nanoparticles.

Also, Nosrati et al. [45] used Bi_2_S_3_ NPs that targeted folate compared to the Cheng et al. study [28], which used non‐targeted Bi_2_S_3_ nanoparticles at the same doses and concentrations, the survival fraction when using targeted nanoparticles was lower, which indicating a greater rate of cellular uptake of nanoparticles through receptors. Consequently, in the study of Song et al. [46], which used bismuth NPs targeted with Arginyl‐glycyl‐aspartic acid (RGD) peptide (8 ppm) compared to the control group in the same doses, the survival fraction was reduced by approximately 50%. This is due to the high uptake of cells from NPs that targeted with RGD peptide, which can target alpha‐v beta‐3 (αvβ3) integrin receptors that are present in large numbers in uterine cancer tissue, while due to the normal tissue receptors of these receptors. In addition, these receptors do not exist on normal tissue and the amount of uptake of these tissues from the nanoparticles reduced. This leads to targeted nanoparticles acting specifically and creating high radiosensitivity in the tumour tissue. The research results indicated that, in addition to exploiting Bi NPs as a radiosensitiser and dose enhancement in radiation therapy, they had been identified as valued theranostics agent in medical imaging.

Bismuth‐based nanoparticles show a unique set of features that harvest a relevant interest such as production of active drugs to diagnostic agents and treatment agents. Furthermore, BiNPs have been deeply investigated by nanoscience and bacterial infections, and as a theranostics application in diagnosis and treatment of cancers [73, 74, 75].

**FIGURE 1:**
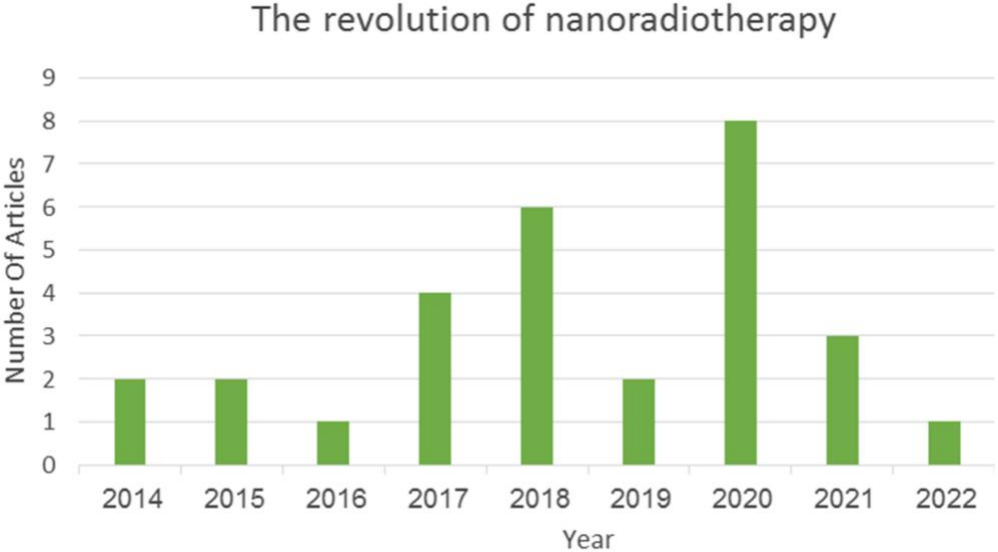
The progress application of BiNPs as radiodensitizers in radiation therapy in the last decade.

This review focussed on the usage of BiNPs in cancer radiation therapy. In recent years, there has been an increasing interest in using metal nanoparticles in medicine for radiation therapy due to their inherent characteristics such as unique physicochemical properties, size, ability to functionalise easily with biomolecules, concentration, low toxicity, and radiosensitiser properties. In addition, methods like IGRT, IMRT, and tomotherapy have been investigated by many researchers. In most studies, gold nanoparticles (AuNPs) were used as a radiosensitiser, but the use of AuNPs has many limitations that make it unable to use extensively in clinical applications [6]. In the current study, the new update of bismuth‐based NPs was investigated in cancer treatment. The limitation of this review is that could not access all newly published papers in this field. Figure 1, showed the last decade progress of the papers number for application of BiNPs as radiodensitizers in radiation therapy.

## 4 CONCLUSIONS AND FUTURE DIRECTIONS

In modern radiation therapy, using metallic nanoparticles because of their high atomic numbers, which highly contributes to interaction with the photons. Bismuth‐based NPs with high atomic number, low cost, availability, low toxicity, and easy coating with other compounds is an attractive radiosensitiser in cancer radiotherapy. BiNPs which known as a radiosensitiser in cancer therapy is one of the ways for future research and approaches as theranostic agents in clinical applications.

This review provides a broad overview on the applicability of BiNPs compounds to increase the efficiency of radiation therapy. Indeed, BiNPs do not have the limitations of gold nanoparticles, and according to this review, bismuth‐based NPs is more effective to annihilate tumor cells than that of gold NPs. However, further studies on toxicity, tumor accumulation, circulation, and studies under in vitro and in vivo conditions are need to be performed to the utility of Bi‐based NPs as a potentiate radiosensitizer into clinical applications. Overall, this review supports the literature for using BiNPs as a suitable platform to improve the quality of radiotherapy and to guide prospective works for their capabilities for cancer treatment in the future.

## AUTHOR CONTRIBUTIONS

Conceptualisation: Daryoush Shahbazi‐Gahrouei, Yazdan Choghazardi, Arezoo Kazemzadeh, Paria Naseri; Methodology: Yazdan Choghazardi, Arezoo Kazemzadeh, Paria Naseri, Saghar Shahbazi‐Gahrouei; Validation: Daryoush Shahbazi‐Gahrouei, Yazdan Choghazardi, Arezoo Kazemzadeh, Paria Naseri, Saghar Shahbazi‐Gahrouei; Investigation: Daryoush Shahbazi‐Gahrouei, Yazdan Choghazardi, Arezoo Kazemzadeh, Paria Naseri, Saghar Shahbazi‐Gahrouei; resources, Daryoush Shahbazi‐Gahrouei; Data curation: Daryoush Shahbazi‐Gahrouei; Writing—original draft preparation: Yazdan Choghazardi, Arezoo Kazemzadeh, Paria Naseri; Writing—review and editing: Daryoush Shahbazi‐Gahrouei, Saghar Shahbazi‐Gahrouei; Supervision: Daryoush Shahbazi‐Gahrouei; Project administration: Daryoush Shahbazi‐Gahrouei; Funding acquisition: Daryoush Shahbazi‐Gahrouei. All authors have read and agreed to the published version of the manuscript.

## CONFLICTS OF INTEREST STATEMENT

The authors declare that they have no conflicts of interest.

## INSTITUTIONAL REVIEW BOARD STATEMENT

This article does not contain any studies with human participants or animals performed by any of the authors.

## Data Availability

The data presented in this study are available on request from the corresponding author.
